# Primary Cutaneous Mucinous Carcinoma of the Nasolabial Fold: A Case Report Highlighting the GATA3, Estrogen, and Progesterone Receptor Diagnostic Pitfall

**DOI:** 10.7759/cureus.102543

**Published:** 2026-01-29

**Authors:** Pritam Ray, Pallabika Mandal, Sanjay Dave

**Affiliations:** 1 Histopathology, Peerless Hospital, Kolkata, IND; 2 Oncosurgery, Peerless Hospital, Kolkata, IND; 3 Pathology, Peerless Hospital, Kolkata, IND

**Keywords:** adnexal neoplasms, diagnostic pitfall, gata3, nasolabial fold, primary cutaneous mucinous carcinoma

## Abstract

Primary cutaneous mucinous carcinoma (PCMC) is an exceptionally rare adnexal malignancy of sweat gland origin, with an age-adjusted incidence of approximately 0.04 per 100,000 person-years. We report a rare occurrence of PCMC on the right nasolabial fold of a 55-year-old male. To the best of our knowledge, based on a review of existing literature, this represents the first reported case of PCMC specifically involving the nasolabial fold within the Indian subcontinent. The lesion initially presented as a slow-growing subcutaneous swelling, mimicking benign entities. Histopathological analysis following excisional biopsy revealed neoplastic nests suspended within expansive lakes of extracellular mucin, partitioned by thin fibrous septa. Immunohistochemical (IHC) profiling demonstrated that the tumor cells were strongly positive for CK7 and EMA and also showed diffuse expression of GATA3, estrogen receptor (ER), and progesterone receptor (PR). This specific immunophenotype - particularly the GATA3 positivity - presents a profound diagnostic trap, as it closely mimics the profile of metastatic mucinous carcinoma of the breast. To navigate this *GATA3/ER/PR trap*, a comprehensive systemic evaluation was mandated, including whole-body positron emission tomography-computed tomography (PET-CT) and mammography, which revealed no evidence of an extracutaneous primary malignancy. This confirmed the diagnosis of PCMC, an indolent but locally aggressive tumor. The patient was managed with wide local excision and remains recurrence-free at one-year follow-up. This case emphasizes that while GATA3, ER, and PR are traditionally associated with mammary origin, their expression in primary cutaneous adnexal tumors is a critical pitfall. Clinicians and pathologists must integrate targeted IHC with rigorous systemic imaging to differentiate PCMC from metastatic disease, ensuring appropriate surgical management and avoiding unnecessary systemic therapy.

## Introduction

Primary cutaneous mucinous carcinoma (PCMC) is an exceptionally rare, low-grade adnexal neoplasm with an estimated age-adjusted incidence of 0.04 cases per 100,000 person-years [1]. Hypothesized to arise from eccrine or apocrine sweat gland epithelium, this tumor poses a significant diagnostic challenge due to its striking morphologic and immunohistochemical similarity to metastatic mucinous carcinoma from internal sites. First described by Lennox et al. in 1952 [2]. This indolent malignancy typically affects individuals in their sixth or seventh decades of life [1]. While global literature suggests a slight male predominance, the most striking clinical feature is its anatomical predilection for the head and neck, particularly the periorbital region and eyelids [3,4]. In the Indian subcontinent, PCMC remains a diagnostic rarity; the available literature consists primarily of sporadic case reports from tertiary centers, often involving facial or axillary sites [1,5,6].

## Case presentation

A 55-year-old man presented in the surgery OPD with a painless, slowly enlarging subcutaneous nodule on the right nasolabial fold, present for approximately one year. Physical examination revealed a firm, non-tender, flesh-colored nodule measuring 1.5 × 1.0 cm, with an intact overlying epidermis. The clinical differential diagnosis included benign entities as well as malignant possibilities. Given the indeterminate clinical features and broad differential diagnosis, an excisional biopsy was judiciously performed for definitive histopathologic diagnosis and potential therapeutic benefit.

**Figure 1:**
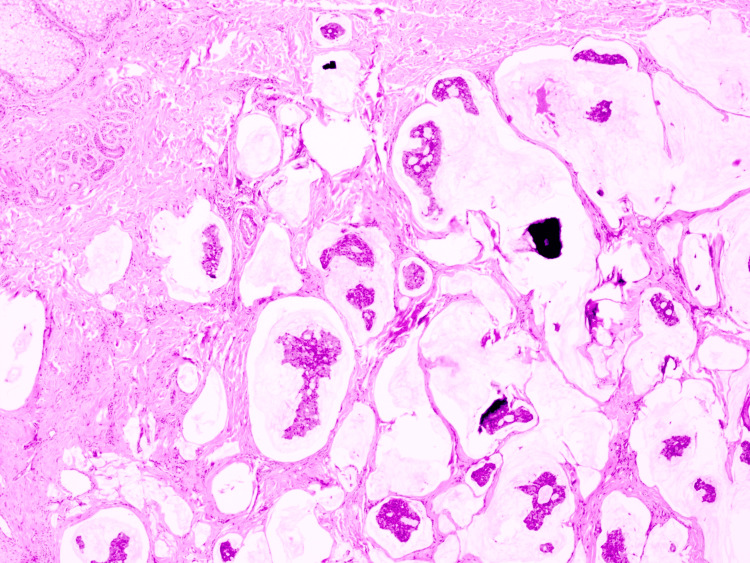
H&E stain (100x). Dermal tumor composed of floating nests, cords, and individual tumor cells within abundant pools of extracellular mucin, compartmentalized by thin fibrous septa. H&E, hematoxylin and eosin

**Figure 2:**
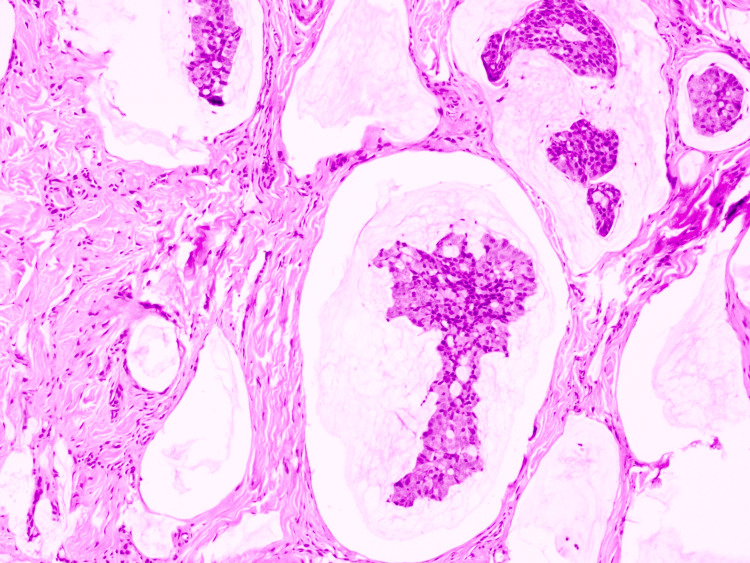
H&E stain (400x). Individual tumor cells exhibited mild cytologic atypia with uniform basaloid features and occasional ductal differentiation. H&E, hematoxylin and eosin

**Figure 3:**
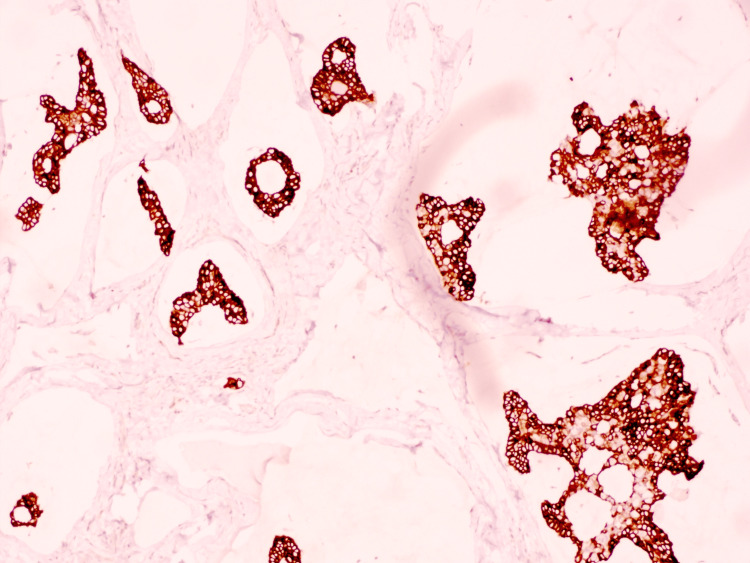
CK7: Tumor cells show strong, diffuse positivity.

**Figure 4:**
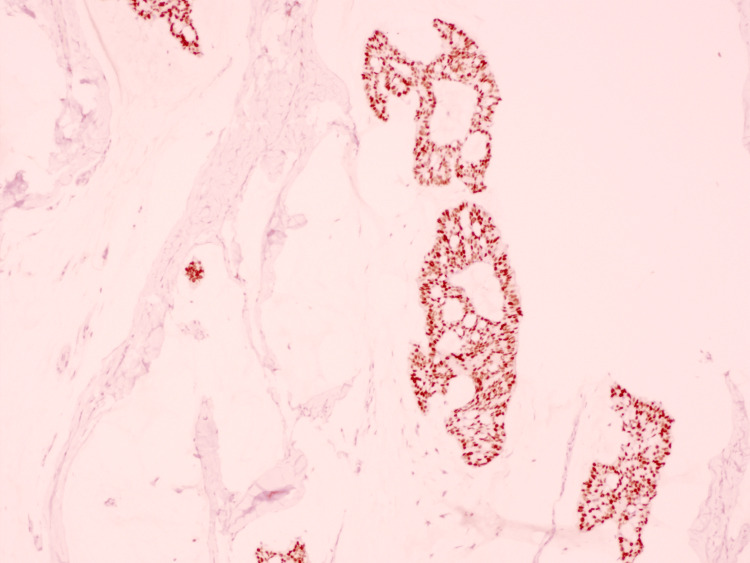
GATA3: Tumor cells show strong nuclear positivity.

**Figure 5:**
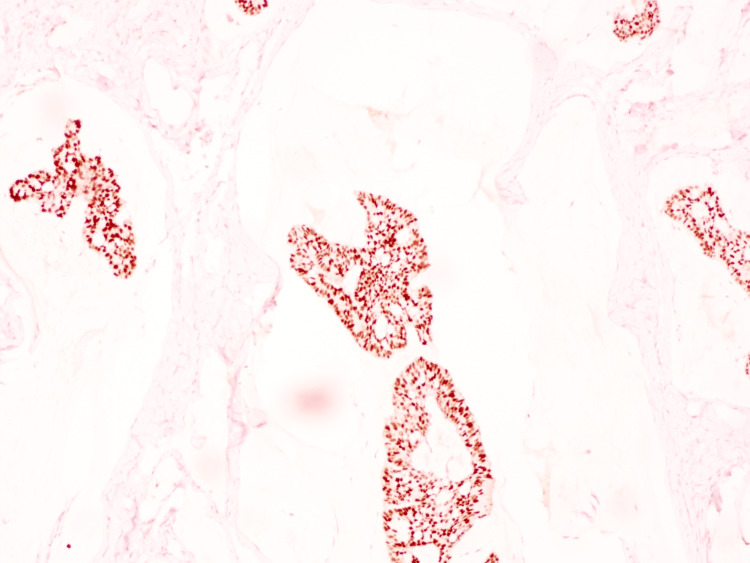
Estrogen receptor (ER): Tumor cells demonstrate strong nuclear positivity.

**Figure 6:**
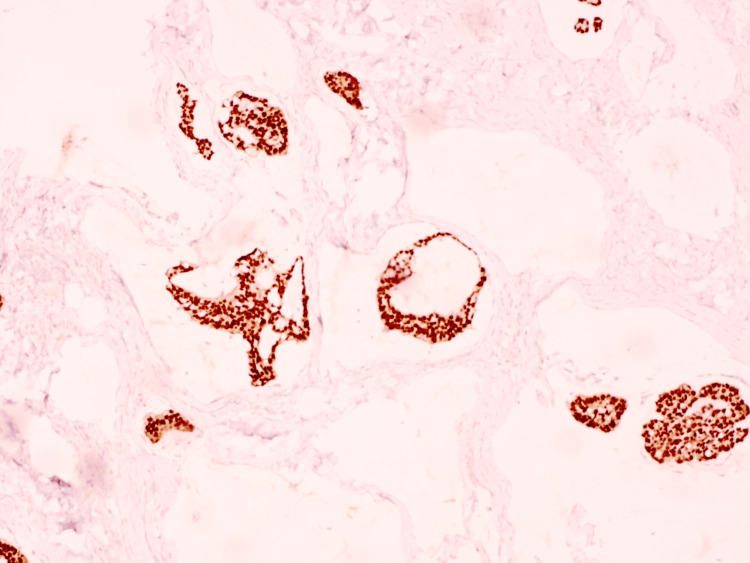
Progesterone receptor (PR): Tumor cells demonstrate strong nuclear positivity.

On histopathologic examination, the biopsy demonstrated a dermal tumor composed of floating nests, cords, and individual tumor cells within abundant pools of extracellular mucin, compartmentalized by thin fibrous septa (Figure 1). The individual tumor cells exhibited mild cytologic atypia with uniform basaloid features and occasional ductal differentiation (Figure 2). Immunohistochemical studies showed tumor cell positivity for CK7 (Figure 3), EMA, GATA3 (Figure 4), ER (Figure 5), and PR (Figure 6), with negativity for CK20, synaptophysin, chromogranin-A, and p63. Tumor islands floating in mucin pools with expression of ER, PR, and GATA3 - markers frequently positive in both primary cutaneous mucinous carcinoma and mammary mucinous carcinoma, representing a well-established diagnostic mimicry that precludes reliable distinction on histopathological and immunohistochemical grounds alone.

To exclude an occult extracutaneous primary, comprehensive systemic staging was undertaken, including positron emission tomography-computed tomography/PET-CT and mammography, both of which revealed no evidence of internal malignancy or metastatic disease. These integrated clinicopathologic and radiographic findings confirmed the diagnosis of primary cutaneous mucinous carcinoma. The patient underwent wide local excision with intraoperative frozen section control, which confirmed clear surgical margins. No adjuvant therapy was required. The patient has remained recurrence-free at the one-year follow-up at the time of reporting; however, given the known potential for late local recurrence in this malignancy, rigorous long-term surveillance is planned.

## Discussion

The diagnosis of PCMC relies heavily on characteristic histomorphology. The tumor typically presents as a lobulated dermal mass composed of small nests, cords, or individual neoplastic cells floating within expansive pools of extracellular mucin [7]. These mucinous lakes are partitioned by delicate fibrous septa. While the presence of an in situ component - such as endocrine mucin-producing sweat gland carcinoma (EMPSGC) - is considered a definitive clue for primary cutaneous origin, it is frequently absent in many cases, including ours [3,8].

The primary diagnostic challenge in PCMC is its remarkable immunohistochemical (IHC) and morphological overlap with metastatic mucinous carcinoma, most commonly from the breast. The IHC profile in this case - CK7+, GATA3+, ER+, and PR+ - is a classic diagnostic pitfall. While GATA3 is a highly sensitive marker for mammary origin, recent evidence confirms its expression in a wide variety of cutaneous adnexal tumors [7,8]. Similarly, ER and PR positivity is common in both PCMC and mammary mucinous carcinoma. The absence of CK20 and neuroendocrine markers (Synaptophysin/Chromogranin-A) effectively helped exclude most GI and primary neuroendocrine origins in our patient [9].

Because IHC cannot definitively distinguish PCMC from a mammary metastasis, a diagnosis of exclusion remains the standard of care. This necessitates a rigorous systemic workup, including PET-CT and, in male patients, clinical breast examination or mammography to rule out occult primary mammary carcinoma [3,7]. In the Indian context, where advanced diagnostics may not be uniformly accessible, the integration of targeted imaging with histopathology is vital to prevent misdiagnosis and inappropriate systemic therapy.

Clinically, PCMC presents as a slow-growing, painless, flesh-colored to bluish nodule that frequently mimics benign conditions. Our observation of its low metastatic potential (approximately 10-13%) but high local recurrence rate (up to 34%) is consistent with the findings of large meta-analyses and SEER database studies, such as those by Kamalpour et al. and Rismiller et al. [1,6]. While nearly 75% of PCMC cases involve the head and neck - predominantly the periorbital region - primary involvement of the nasolabial fold is remarkably rare [1,6,8]. Within the Indian subcontinent, where PCMC itself remains a diagnostic rarity, our case contrasts with the more typical periorbital and axillary presentations reported by Chauhan et al. and Mardi et al. [3,4]. To our knowledge, this represents the first documented case of PCMC arising specifically in the nasolabial fold. Management at our center involved wide local excision with clear margins. Although Mohs micrographic surgery is often advocated for tissue-sparing in cosmetically sensitive areas, conventional wide excision remains a highly effective and standard approach in many tertiary Indian centers, yielding excellent long-term outcomes provided that margin clearance is histologically confirmed [4,5].

## Conclusions

PCMC is a rare but significant diagnostic entity that requires a high index of suspicion. Our case highlights the critical importance of recognizing the *GATA3/ER/PR* trap, where IHC markers traditionally associated with the breast are expressed in a primary skin adnexal tumor. This report enriches the sparse Indian literature on PCMC and underscores the necessity of a multidisciplinary approach - combining meticulous pathology with systemic imaging - to ensure a definitive diagnosis and a favorable surgical outcome.
